# Avalanche Analysis from Multielectrode Ensemble Recordings in Cat, Monkey, and Human Cerebral Cortex during Wakefulness and Sleep

**DOI:** 10.3389/fphys.2012.00302

**Published:** 2012-08-03

**Authors:** Nima Dehghani, Nicholas G. Hatsopoulos, Zach D. Haga, Rebecca A. Parker, Bradley Greger, Eric Halgren, Sydney S. Cash, Alain Destexhe

**Affiliations:** ^1^Laboratory of Computational Neuroscience, Unité de Neurosciences, Information et Complexité, CNRSGif-sur-Yvette, France; ^2^Department of Organismal Biology and Anatomy, Committee on Computational Neuroscience University of ChicagoChicago, IL, USA; ^3^Interdepartmental Program in Neuroscience, University of Utah, Salt Lake CityUT, USA; ^4^Department of Bioengineering, University of Utah, Salt Lake CityUT, USA; ^5^Multimodal Imaging Laboratory, Departments of Neurosciences and Radiology, University of California San DiegoLa Jolla, CA, USA; ^6^Department of Neurology, Massachusetts General Hospital and Harvard Medical SchoolBoston, MA, USA

**Keywords:** criticality, self-organization, brain dynamics, scale invariance, complexity, power-law

## Abstract

Self-organized critical states are found in many natural systems, from earthquakes to forest fires, they have also been observed in neural systems, particularly, in neuronal cultures. However, the presence of critical states in the awake brain remains controversial. Here, we compared avalanche analyses performed on different *in vivo* preparations during wakefulness, slow-wave sleep, and REM sleep, using high density electrode arrays in cat motor cortex (96 electrodes), monkey motor cortex and premotor cortex and human temporal cortex (96 electrodes) in epileptic patients. In neuronal avalanches defined from units (up to 160 single units), the size of avalanches never clearly scaled as power-law, but rather scaled exponentially or displayed intermediate scaling. We also analyzed the dynamics of local field potentials (LFPs) and in particular LFP negative peaks (nLFPs) among the different electrodes (up to 96 sites in temporal cortex or up to 128 sites in adjacent motor and premotor cortices). In this case, the avalanches defined from nLFPs displayed power-law scaling in double logarithmic representations, as reported previously in monkey. However, avalanche defined as positive LFP (pLFP) peaks, which are less directly related to neuronal firing, also displayed apparent power-law scaling. Closer examination of this scaling using the more reliable cumulative distribution function (CDF) and other rigorous statistical measures, did not confirm power-law scaling. The same pattern was seen for cats, monkey, and human, as well as for different brain states of wakefulness and sleep. We also tested other alternative distributions. Multiple exponential fitting yielded optimal fits of the avalanche dynamics with bi-exponential distributions. Collectively, these results show no clear evidence for power-law scaling or self-organized critical states in the awake and sleeping brain of mammals, from cat to man.

## Introduction

Self-organized criticality (SOC) is a dynamical state of a system which maintains itself at (or close to) a phase transition point. This family of systems were initially described by Bak et al. (1987), and have been found in many natural systems (reviewed in Bak, 1996; Jensen, 1998). SOC systems are characterized by scale invariance, which is usually identified as a power-law distribution of characteristics of the system’s dynamics such as event size or the waiting time between events. The temporal fingerprint of SOC systems is often 1/*f* or 1/*f* ^2^ noise. These features are interesting because they show the presence of long-lasting or long-range correlations in the system.

The dynamics of SOC systems are structured as “avalanches” of activity, separated by silent periods. Avalanche sizes are typically distributed as a power-law, where the probability of occurrence *p*(*x*) of a given avalanche size *x* scales as:

$$p\left(x\right)∼x^{-\alpha },$$

where α is the scaling exponent of the distribution.

SOC systems have been observed in many different natural phenomena, from sandpiles, to rice piles, in forest fires, and earthquakes (Bak and Paczuski, 1995; Bak, 1996; Frette et al., 1996; Jensen, 1998; Malamud et al., 1998; Peters and Neelin, 2006). Evidence of SOC was also demonstrated in circuits of neurons *in vitro* (Beggs and Plenz, 2003), where network activity was found to alternate between active and quiescent periods, forming “neuronal avalanches.” The presence of avalanches, although clear *in vitro*, is more controversial *in vivo*. Since power-laws fit neuronal avalanches better than other alternative probability distributions (Klaus et al., 2011), their presence has been taken as evidence for neuronal avalanches *in vivo*. In anesthetized cats (Hahn et al., 2010) and awake monkeys (Petermann et al., 2009), power-law distributed avalanches have been found in the peaks of local field potentials (LFP). However, LFP peaks are only statistically related to neuronal firing. In a study on awake and naturally sleeping cats, no sign of avalanches were found in neuronal firing (Bedard et al., 2006), and the apparent power-law scaling of LFP peaks could be explained as an artifact induced by the thresholding procedure used to detect LFP peaks. Previous studies have shown that even purely stochastic processes can display power-law scaling when subjected to similar thresholding procedures (Touboul and Destexhe, 2010). It was also stressed that power-law statistics can be generated by stochastic mechanisms other than SOC (Giesinger, 2001; Chialvo, 2010; Touboul and Destexhe, 2010). Similarly, if exponentially growing processes are suddenly killed (or “observed”), a power-law at the tail ends will emerge (Reed and Hughes, 2002). This case, would be similar to a non-stationary Poisson processes, or combining Poisson processes at different rates, a situation that is likely to happen in the nervous system. Such scenarios can give rise to spurious power-laws.

These contrasting results correspond to different preparations and recording techniques, single units or LFPs, or different species, so that it is difficult to compare them. In the present paper, we attempt to overcome these shortcomings by providing a systematic analysis of both units and LFPs for different species and different brain states.

## Materials and Methods

### Recordings

#### Cat

Recordings of local field potentials (LFPs) and action potentials (APs) were obtained from motor cortex in 2 felines (M1 and approximately hindlimb region). Commercially obtained 96 electrode sputtered iridium oxide film arrays (Blackrock Microsystems, Inc., Salt Lake City, UT, USA) were chronically implanted and recordings were performed in the awake, unrestrained feline (as described in Parker et al., 2011). Electrodes on the array were arranged in a square with 400 micron spacing and 1 mm shank length. LFPs and APs were recorded using a Cerebus data acquisition system (Blackrock Microsystems). Spike sorting on AP data was performed using the t-dist EM algorithm built into Offline Sorter (Plexon, Inc.). All animal procedures were performed in accordance with University of Utah Institutional Animal Care and Use Committee guidelines.

We also compared these data with previously published multielectrode data on cat parietal cortex (Destexhe et al., 1999). In this case, a linear array of 8 bipolar electrodes (separated by 1 mm) was chronically implanted in cortical area 5–7, together with myographic and oculographic recordings, to insure that brain states were correctly discriminated (quiet wakefulness with eyes-open, slow-wave sleep, REM sleep). Throughout the text, this cat will be referred to as “cat iii” LFP signals were digitized offline at 250 Hz using the Igor software package (Wavemetrics, OR, USA; A/D board from GW Instruments, MA, USA; low-pass filter of 100 Hz). Units were digitized offline at 10 kHz, and spike sorting and discrimination was performed with the DataWave software package (DataWave Technologies, CO, USA; filters were 300 Hz high-pass and 5 kHz low-pass).

#### Monkey

Recordings from three monkeys were used in this study. Each monkey was chronically implanted with 100-electrode Utah arrays (400 m inter-electrode separation, 1.0 mm electrode length; BlackRock Microsystems, Inc., Salt Lake City, UT, USA). In two monkeys (i) and (ii), we used recordings made during the performance of motor tasks. The motor tasks involved moving a cursor to visually presented targets in the horizontal plane by flexing and extending the shoulder and elbow of the arm contralateral to the cerebral hemisphere that was implanted. In monkey (iii), sleep recordings were used to test avalanche dynamics. Monkey i was implanted with one 96 electrode array in primary motor cortex (MI) and a second 96 electrode array in dorsal premotor cortex (PMd) from which recordings were made on 64 electrodes in each cortical area. Monkey ii had an array implanted in MI from which 96 electrodes were recorded and monkey iii had two arrays in MI and PMd from which 96 electrodes were recorded in PMd cortex and 32 electrodes were recorded in MI area. During a recording session, local field potential (LFP) signals were amplified (gain, 5000), band-pass filtered (0.3–250 or 0.3–500 Hz), and recorded digitally (14-bit) at 1 kHz per channel To acquire extracellular action potentials, signals were amplified (gain, 5000), band-pass filtered (250–7.5 kHz) and sampled at 30 kHz per channel. For each channel, a threshold was set above the noise band: if the signal crossed the threshold, a 1.6-ms duration of the signal – as to yield 48 samples given a sampling frequency of 30 kHz – was sampled around the occurrence of the threshold crossing and spike-sorted using Offline Sorter (Plexon, Inc., Dallas, TX, USA). All of the surgical and behavioral procedures performed on the non-human primates were approved by the University of Chicagos IACUC and conform to the principles outlined in the Guide for the Care and Use of Laboratory Animals (NIH publication no. 86–23, revised 1985).

#### Human

Recordings were obtained from two patients with medically intractable focal epilepsy using NeuroPort electrode array as discussed previously (Truccolo et al., 2010; Peyrache et al., 2012). The array, 1 mm in length, was placed in layers II/III of the middle temporal gyrus with informed consent of the patient and with approval of the local Institutional Review Board in accordance with the ethical standards of the Declaration of Helsinki. This array is silicon-based, made up of 96 microelectrodes with 400-μm spacing, covering an area of 4 mm × 4 mm. Since the corners are omitted from the array, the furthest separated contacts are 4.6 mm apart. Data were sampled at 30 kHz (Blackrock Microsystems, Salt Lake City, UT, USA). The continuous recording was downsampled to 1250 Hz to obtain LFPs. The dataset we analyzed was devoid of any form of identifiable epileptic activity (such as interictal spikes), and there was no seizure in the analyzed dataset. The implantation site was included in the therapeutic resection in both patients. For details on spike sorting, see Peyrache et al. (2012).

### Avalanche detection

Avalanches are defined by temporally contiguous clusters of activity among the different electrodes, separated by periods of silence. Either trains of neuronal action potentials (spikes) or LFP peaks can be analyzed for the occurrence of avalanches. There are two empirical limits on bin duration. The smallest bin size is set by the duration of the action potential. The upper boundary, is limited by how many unique values of the aggregate ensemble activity occur in a window. When the number of unique values approaches 1, avalanche loses its definition, because there is no silent period left. In the cat data, where there are 160 cells, we reach this limit at a bin-width of 16 ms. So, we have stayed within the 1- to 15-ms regime in which an avalanche could be well defined.

#### Spike avalanche

**Figure 1:**
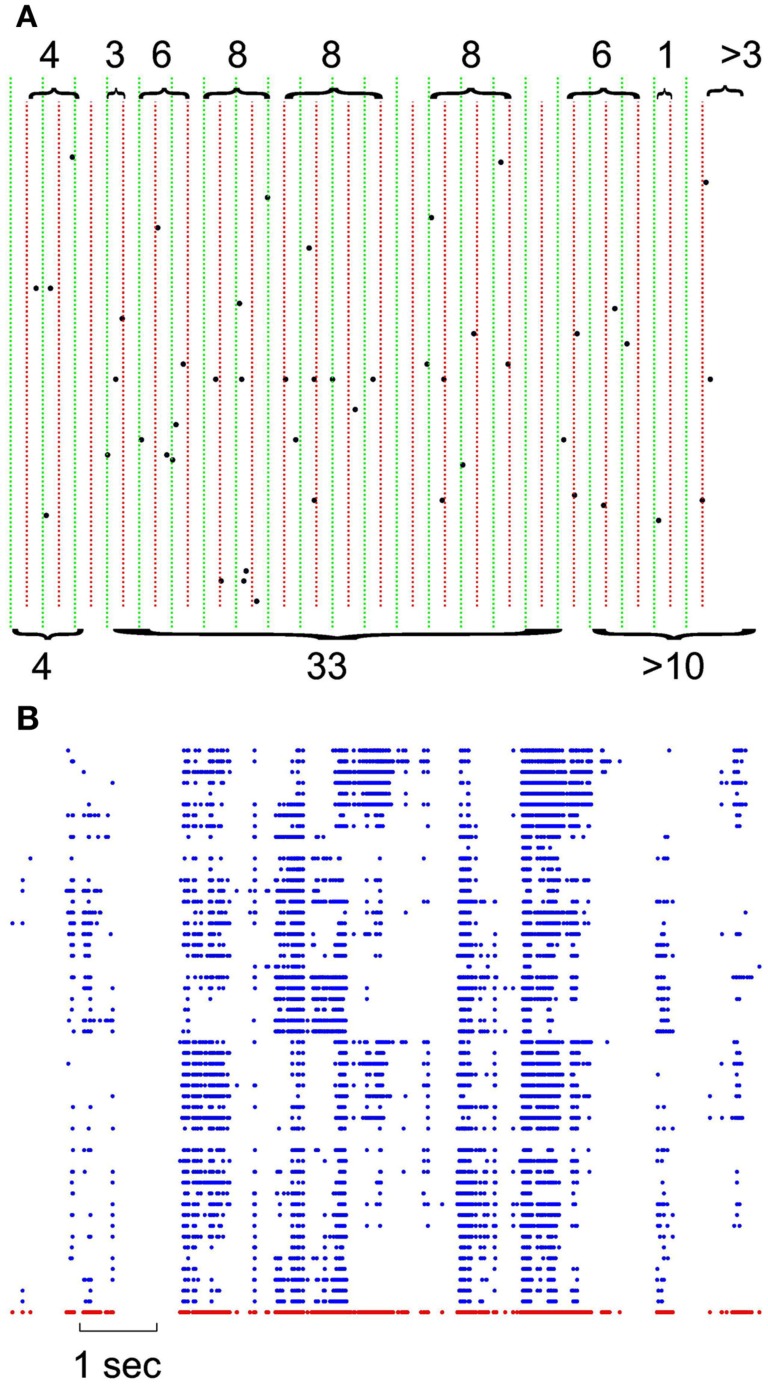
**Definition of avalanches**. **(A)** Comparison of avalanche definition for 8 vs. 16 ms binning; green vertical lines define the boundaries of 16 ms binning; naturally, each 16 ms bin is composed of 2 independent 8 ms bin (depicted with red dotted lines). Accolades point to the avalanches, separated by quiescent periods. Top, 8 ms avalanches and their sizes, bottom, 16 ms avalanches and their corresponding size. Please note that last avalanche continues after of the limits in this figure. **(B)** Negative local maxima obtained from the grid of electrodes for a period of 10 s. Each row represents negative maxima of a single LFP channel of a selected threshold level ≥1.75 × STD of the normalized LFP. The red dots in the bottom refer to ensemble presence of nLFP maxima.

In each set of recordings, regardless of the spatial location of a given electrode in the multielectrode array, its spiking activity was put in the same pool with all other spikes recorded from other electrodes of the same array. This ensemble trace was then binned and coarse grained for different δt ranging from 1 to 16 ms in 2 ms steps. This created a series of bins containing the ensemble of activity across all neurons for that δt. The sum of spiking in that bin represents the total bin activity. The sum of all bin activities between two quiescent bins, represents the avalanche size, which was later used for statistical analyses. Notice that in the case of the minimum δt = 1, avalanche size would range between 0 and maximum number of neurons present as this bin approximates the size unity of spiking period. Figure 1A shows the definition of avalanche in spike series from human recordings.

#### LFP avalanche

Each LFP trace was first detrended through a least-squares fit of a straight line to the data and subsequent subtraction of the resulting function from all the sample points. After this detrending removed the mean value or linear trend from a LFP vector, it was then normalized (Z score) to have a common reference frame for discretization across channels, recordings, states, and species. The z-scored LFP, was then discretized through a local maxima peak detection. An optimizing small running average filter was designed and 3 passes of the filter were applied to the data in order to remove small spurious peaks in each LFP deflection. Next, by comparing each element of data to its neighboring values, if that sample of data was larger than both of its adjacent ones, that element was considered as a local peak. Next, all the peaks were sorted in descending order, beginning with the largest peak, and all identified peaks not separated by more than minimum peak distance (of 3 samples) from the next local peak were discarded.

The threshold was fixed and defined as a multiple of the standard deviation (STD) of the LFP signal. Different thresholds were tested, starting at 1.25 × STD and increasing in 0.25 steps up to 5 × STD for both negative and positive maxima. This procedure was realized on each LFP channel, state, species (Figure 1B). Such matrix of discrete events (for a given polarity and a given threshold), was then treated the same way the spike matrix was used to create avalanche vectors of quiescent and active periods.

### LFP peak and spiking relationship

#### Wave-triggered-average (WTA)

We used wave-triggered averaging (WTA) to analyze the differences in the relationships of spikes to nLFP vs. pLFP. In WTA, the individual negative LFP peaks (nLFP) were used to epoch the ensemble spike series. The epoched ensemble spike series were normalized by the number of epochs (triggered by nLFPs). This procedure was performed for the three different thresholds (low, medium, and high) and the results were averaged across these thresholds to obtain cross-threshold WTA percentage firing to quantify the spike-nLFP relationship. An identical procedure was applied to pLFPs. The red and blue solid lines in Figure 6 refer to nLFP-spike and pLFP-spike WTA percentage firing, respectively.

#### Controls and randomization Methods

We used 4 methods of surrogate/randomization in order to evaluate the statistical robustness of the comparative relation of spike-nLFP vs. spike-pLFP. Each of the following 4 methods, was first performed on all 3 chosen thresholds and then the results were averaged to obtain the overall randomization effect.

##### Poisson surrogate data

At the first step, we wanted to test whether the observed nLFP and pLFP differences could be reproduced by surrogate spike series. For this type of control, first, each individual channel’s spike rate was calculated. Then, using a renewal process, a surrogate Poisson spike series for that channel was created (matching the firing rate and duration of the experimental data from that channel). Then, all Poisson spike series (across all channels) were aggregated together to create the ensemble spike series (similar to the experimental data). Next, for each pLFP (or nLFP), the WTA of this Poisson aggregate series was created. This procedure was repeated 1000 times and then averaged across the 1000 trials. The results were close to a constant WTA percent firing and did not fluctuate according to the timing of the peak LFP that was used to epoch each individual WTA event. This control test showed that the simple aggregate of surrogate Poisson spikes can not reproduce the observed relation between nLFP and spikes in the WTA or mimic the behavior of natural peak(positive or negative)-induced percentage firing. This procedure was also repeated with Poisson spikes without a refractory period and provided similar results.

##### Random permutation

In a follow up test, we wanted to verify that randomizing the aggregate spike series by itself can not mimic the observed the LFP-spike relation. For this procedure, we performed a random permutation on the aggregate spike series and then calculated the nLFP(and pLFP)-based WTA. This procedure was repeated 1000 times. The observations are similar to the Poisson randomization, verifying that the nLFP peak is not reproducible by randomization of spikes and the fluctuations of WTA percentage firing are not results of random events.

##### Local jitter randomization of LFP peaks

Next, we wanted to evaluate the effects of randomization based on the statistics of the individual channel’s LFP peak times (before aggregating them into the ensemble LFP peak train). First, each channel’s nLFP IPI (inter-peak-interval) were calculated. Then these IPIs from all channels were put in the same pool and the, 0.25, 0.5, and 0.75 quantiles IPI for the aggregate nLFPs were extracted. Next, we created a normal distribution with 0.5 percentile as the mean, the interquartile range (0.75 − 0.25 quantile) as the standard deviation of the pdf, and N events matching the number of aggregate nLFPs. This set of values, were used to jitter nLFPs in the following manner. Each sample from the aggregates nLFP peak series was shifted according to one drawn sample (without replacement) from the nLFP jitter pool. The direction of the shift was to the right if the drawn jitter value was negative (and to the left for the positive value). The magnitude of the shift was defined by the value of the jitter itself. The same procedure was repeated for pLFPs. The results of this randomization are shown in Figure 6A. As can be appreciated, with this tightly regulated data-driven local randomization, the structure of the WTA is preserved except for the peak curve around 0 for the nLFP case.

##### Fixed-ISI circular shift of spikes

In this procedure, we kept the ISI (inter-spike interval) of the aggregate spike series as well as the IPI (inter-peak intervals) of the nLFP and pLFP intact but randomized the relation between the aggregate spike and aggregate peak series. In each of the 1000 trials, a circular shift with the magnitude chosen randomly between 1 and the range of the ISI, was performed. The results, shown in Figure 6B, show that by destroying the relation between ensemble spikes and ensemble peaks while preserving their internal structure, the observed fluctuations and most importantly, the tightly bound relation of nLFP and spikes, is lost.

### Testing power-law distribution in empirical data

For testing the power-law behavior, usually a simple least square method is applied to fit a power-law on the data. If such fit in a log-log scale, follows a straight line, the slope of the probability density function (PDF) line is taken as the scaling exponent. Such method is widely practiced but is highly inaccurate in its estimation of true existence of power-law in a given dataset. It has been argued that, for obtaining statistically sound results in estimating power-law in empirical data, one has to rely on rigorous statistical methods. In a detailed analysis of the problem (Newman, 2005; Clauset et al., 2009), it was proposed that the cumulative distribution function (CDF) is much more accurate to fit the power-law exponent, as well as to identify if the system obeys a power-law.

If the initial distribution of the PDF is power-law, i.e.,

$$p\left(x\right)=Cx^{-\alpha },$$

then CDF is defined as

$$Pr\left(X>x\right)=C\int_{x}^{inf}\;{x^{\prime }}^{-\alpha }dx^{\prime }=\frac{C}{\alpha -1}x^{-\left(\alpha -1\right)}.$$

Thus, the corresponding CDF also behaves as a power-law, but with a smaller exponent

α-1

being 1 unit smaller than the original exponent (Newman, 2005).

Generally, in fitting the power-law to the empirical data, all the initial values (left hand of the distribution histogram, i.e., smallest sizes of avalanches) are included in the used decades to obtain the slope of the fit (scaling exponent α). The inclusion of these initial parts may cause significant errors, and should be removed (Goldstein et al., 2004; Bauke, 2007; Clauset et al., 2009). Thus, before calculating the scaling exponent, it is essential to discard the values below the lower bound (*X_min_*). It is only above this lower bound that, a linear PDF or CDF can be reliably used for estimation of the scaling exponent. There are different methods for proper estimation of the *X_min_*. We used a Kolmogorov-Smirnov (KS test) optimization approach that searches for the minimum “distance” (D) between the power-law model and the empirical, where for Xi ≥ *X_min_*, “D” is defined as

$$D=max|S\left(x\right)-P\left(x\right)|,$$

*S*(*x*) the CDF of the empirical data and *P*(*x*) the CDF of the best matching power-law model. The *X_min_* value that yields the minimum D, is the optimal *X_min_*. The *X_min_* is used in a maximum likelihood estimate (MLE) of power-law fit to the CDF of the avalanches in order to obtain the scaling exponent. This fitting, however, does not provide any statistical significance on whether the power-law is a plausible fit to the data or not. After the estimation of *X_min_* and the exponent, we generated N (N = 1000) power-law distributed surrogate data with the exact same features of *X_min_* and exponent. Each of these surrogate series are then fitted with power-law and KS-statistics of distance D (to the surrogate power-law), is performed. The fraction of N that the resultant statistics was bigger than the one obtained from the empirical data, comprises the *p*-value. If *p*-value ≤0.1, the power-law is ruled out. However, even if *p*-value is larger than this threshold, the data is not necessarily guaranteed to be generated by a power-law process unless no better distribution is found to estimate the properties of the data. For this, the alternative test was adapted as following.

#### Generating power-law distributed random numbers with high precision

It is essential to use high precision and reliable algorithms to generate random numbers from a given probability distribution; otherwise the statistical tests based on such distributions may be erroneous. For initializing the generator with an “Integer Seed,” we adapted the reliable Mersenne Twister algorithm (known as MT19937AR) with full precision of Mersenne prime (2^19937^ − 1) (Matsumoto and Nishimura, 1998). This algorithm provides a proper method for running Monte Carlo simulations. After initialization, “Transformation algorithm” was used to generate the desired distribution (Press et al., 2007a; Clauset et al., 2009). All the random number generations and analyses were performed on a 16-core Intel 48 GB Linux platform equipped with 448 core Tesla C2050 GPU with double precision of 515 Gflop and single precision of 1.03 Tflops. The custom code was based on Matlab (Mathworks) and CUDA (NVIDIA) wrapper Jacket (Accelreyes) for parallel computing on GPU.

### Alternative fits

The power-law fit was compared with alternative hypotheses to test which distribution best fits the data. The alternatives included exponential distribution (as predicted by a Poisson type stochastic process), “Discretized log-normal distribution” (which is represented as a linear fit in log-normal scale), as well as fit of “Discrete exponential distribution” nature. These fits had two general types of simple exponential, defined as: *f*(*x*) = *a*exp(*bx*) as well as sum of exponential set as: *f*(*x*) = *a*exp(*bx*) + *c*exp(*dx*) In each case, residual analyses, goodness of fit as well as confidence and prediction bounds were used to evaluate the properties of each fit vs. power-law. In case of a good fit model, Residual, defined as the difference between data and fit, should approximate random error and behave randomly.

#### Goodness of fit comparison of exponential models

A measure of “goodness of fit,” *R*-square, is the ratio of the sum of squares of the regression (SSR) and the total sum of squares (SST). This measure, represents the square of the correlation between the observed and predicted response values, and indicates what percentage of the variance of the data is explained by the chosen fit (values of *R*-square range from 0, worst fit, to 1, the best possible fit). If we have SSR as: $SS_{\text{reg}}=\sum_{i}\;{\left({ŷ}_{i}-ȳ\right)}^{2},$ and SSE as: $SS_{\text{err}}=\sum_{i}\;{\left(y_{i}-ŷ\right)}^{2},$ and SST as: $SS_{\text{tot}}=\sum_{i}\;{\left(y_{i}-ȳ\right)}^{2},$ where, $y_{i},ȳ,ŷ$ are the original data values, their mean and modeled values respectively. Then, it follows that:

$$R^{2}=SS_{\text{reg}}∕SS_{\text{tot}}=1-\frac{SS_{\text{err}}}{SS_{\text{tot}}}.$$

Correction by “total degree of freedom” and “error degree of freedom,” defines adjusted *R*-square:

$${\bar{R}}^{2}=1-\left(1-R^{2}\right)\frac{N-1}{N-M-1}=1-\frac{SS_{\text{err}}}{SS_{\text{tot}}}\frac{df_{t}}{df_{e}}.$$

where “N” is the sample size, and “M” is the number of fitted coefficients (excluding constants). Usage of ${\bar{R}}^{2}$ in the comparison of “simple exponential” and “sum of exponential” is warranted by the fact that by an increase in the fitted number of the components, from one model to the other, the degrees of freedom changes. Both *R*^2^ and ${\bar{R}}^{2}$ measures were estimated through non-linear least square optimization of exponential curve fitting. In the optimization process for estimating the coefficients of the models, we adapted Levenberg-Marquardt algorithm with a tolerance of 10^−8^ (Press et al., 2007b).

#### Test of linearity in log-normal scale

Linearity in log-normal scale, is a hallmark of an exponential family process. In order to test the linearity of the PDF in log-normal scaling, we used Root mean square error (RMSE), $\text{RMSE}\left(\hat{\theta }\right)=\sqrt{\text{MSE}\left(\hat{\theta }\right)}$ where MSE is: $\frac{SS_{\text{err}}}{df_{e}}.$ This measure ranges from 0 to 1, where closer value to 0 is an indicator of a better fit.

This test was performed by fitting *y* = *log*[*P*(*x*)] with a linear least square first degree polynomial. As shown in Figure 13C, sometimes, the initial values in the left tail may slightly deviate from a simple 1st degree polynomial. Therefore, we tested whether the linearity was improved or worsened when the data range was reduced to above some *X_min_*. For doing so, we adapted a more stringent regression, bi-square robust 1st degree polynomial (Press et al., 2007b). This method is an iteratively reweighted least-squares, based on ${\bar{R}}^{2},$ and assigns less weight to the values farther from the line. This procedure was repeated after excluding consequent single values from the left tail (up to 20% of the points). For each new shortened series, the RMSE (based on bi-square method) was re-calculated. The rational behind using RMSE for testing the linearity range in these datasets (with variable N) is that when a distinct point is removed from the dataset, 2 other reductions follow: (a) the sum of squares and (b) degrees of freedom. Thus, if after limiting the range, the error remains the same, *SS*_err_ would increase. Similarly, when the error is significantly reduced, *SS*_err_ would increase. Therefore, any change in the error, should only be considered significant if it is compensated by the amount of change in the degree of freedom. For quantifying this, we defined two measures for linearity improvement after limiting the data above *X_min_*. The first measure, “overall RMSE change” (oRMSE), was defined as:

$$oRMSE_{i}=\frac{RMSE_{n}-RMSE_{n-i}}{RMSE_{n}}*100.$$

In parallel, “relative RMSE change” (rRMSE), was defined as:

$$rRMSE_{i}=\frac{RMSE_{n-i+1}-RMSE_{n-i}}{RMSE_{n}}*100,$$

where *RMSE_n_* was the RMSE of the full length data. Next standard deviation of the, these measures were normalized to their maximum (*noRMSE* and *nrRMSE*) and a 3rd dimension was created by the distance of each pair (*noRMSE_i_*, *nrRMSE_i_*), from the geometrical diagonal defined as

$$D=\frac{det\left[\left(Q2-Q1\right)\cdot \left(P-Q1\right)\right]}{∥\left(Q2-Q1\right)∥}$$

where P was the coordinates of a point (*noRMSE_i_*, *nrRMSE_i_*) while Q1 = [0 0] and Q2 = [1 1] were the vertices of the geometrical diagonal of the RMSEs pair space. The point that had the maximum “(1 − *D_i_*) + *noRMSE_i_* + *nrRMSE_i_*” (this value can range between 0 and 3), was taken as the optimal linearizing shortening index (*X_min_*; Figure 13D). Next, we fitted all data ranges (from *N* sample points to *N* − *X_min_*) with the two exponential models as described above.

## Results

In this study, we used data from multielectrode recordings in 3 species: cat motor cortex (cats i and ii with a 96 channel multielectrode array in primary motor cortex, hindlimb area), cat parietal cortex (cat iii, 8 bipolar electrodes), monkey motor cortex (three monkeys with a 64 or 96 recordings from 96 channel multielectrode arrays in motor and/or premotor cortex), and humans (2 patients with a 96 multielectrode array in middle temporal gyrus). In the following, we briefly address definition of avalanche, then describe the results of power-law analyses on spike avalanche, state-dependence, regional differences, and polarity-dependence of LFP maxima avalanche. At the end, we briefly discuss alternative fits to the data.

### Avalanche definition

Figure 1 illustrates the definition of avalanche for discrete (spike) and continuous (LFP) data, as they are used in this study. For both spikes and LFP, we used bins of 1–15 ms (in 2 ms steps) for defining the quiescent vs. active periods. Avalanches are defined by contiguous bins of non-zero activity, separated by periods of quiescence (empty bins). The size of the avalanche is defined as the sum of all activities (spikes or LFP peaks) within that active period. Thus, the avalanches depend on the bin size (as illustrated in Figure 1A for spikes). For LFPs, we first discretized the continuous data based on its local maxima. Both positive and negative maxima were examined in our study. For each polarity, 17 levels of thresholds were chosen (see Methods for details). After discretization, the obtained matrix (Figure 1B) was used for the same binning and avalanche definition as used for spike series.

### Power-law fit

It has been shown that that CDF provides a better measure than PDF as it avoids erroneous measures at the far end of the distribution tail of probability curve (Newman, 2005; Clauset et al., 2009). It is also necessary to exclude the values below the valid lower bound, or else the calculated coefficient could be highly biased (Clauset et al., 2009). In each of the following estimates of power-law distribution, based on the methods described previously, we adapted the following steps on analyzing the CDF of avalanches: Values above a given *X_min_* are used in a maximum likelihood estimate (MLE) of the exponent α. For each CDF, the proper lower bound of *X_min_* is selected using a KS test. We also used 1000 semi-parametric repetitions of the fitting procedure for obtaining estimates of uncertainty and goodness of fit.

### Avalanche analysis from spikes

Next, we studied whether the spike avalanches follow power-law distributions.

#### Avalanche analysis in wakefulness

**Figure 2:**
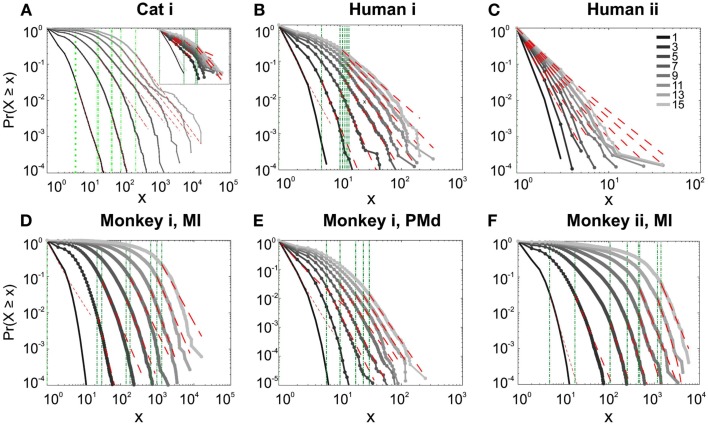
**Avalanche analysis on spiking activity during wakefulness**. In idle awake **(A)**. Cat i (96 electrode array) and Cat iii (inset, 8 electrode array). **(B)** Human i (96 electrode array). **(C)** Human ii (96 electrode array). Different line colors refer to different bin sizes as shown in the legend. The lower bound (*X_min_*, shown in green dotted line), shows that the CDF of avalanche size, only partially, may follow power-law distribution. Even in such cases, the exponents had very high values, well above the criticality regime that is hypothesized for 1/*f* noise. **(D–F)** Show the same type of curves for monkeys engaged in cognitive motor task (96 electrode array; augmented with a 64 electrode array). Same pattern is observed; it also seems MI has slightly higher values than PMd in the plausible power-law regime. For the mean/STD exponent values, see Tables 1 and 2.

We first studied avalanche dynamics in awake resting recordings from cats and humans. As depicted in Figure 2, neither of these species, showed a dominant power-law behavior in their spike avalanche size distribution. The average scaling exponent of awake recordings for the decades that could be considered to follow power-law (i.e., >*X_min_*), was to high to be related to SOC systems (see Tables 1 and 2; Figures 2i,ii,iii). These values not only are distant from those of 1/*f* noise, but also only apply to partial parts of the CDF (cumulative distribution function) of avalanche sizes. These lack of clear power-law characteristics is shown with *X_min_* lower boundary (green dotted lines in Figure 2). Only values above *X_min_* could “statistically” follow a power-law regime and as mentioned, even in those cases, the exponent values were too high to be considered a signature of SOC systems. It is important to note that the CDF representation is cumulative, and thus the left tail is not excluded from the data but its influence is shifted to the right (see details in Clauset et al., 2009; see also Methods).

**Table 1:** **Summary spike avalanche**.

Species	Loc	State	CDF exponent	Pval	gof
Monkey i	MI	Awake	3.4413 ± 0.7616	0.0419 ± 0.1152	0.0442 ± 0.0216
Monkey i	Pmd	Awake	4.1660 ± 0.6590	0.1130 ± 0.2140	0.0180 ± 0.0050
Monkey ii	MI	Awake	4.6250 ± 0.4730	0.4550 ± 0.3600	0.0330 ± 0.0120
Monkey iii	MI	SWS	4.5560 ± 0.7980	0.0030 ± 0.0100	0.0220 ± 0.0080
Monkey iii	Pmd	SWS	3.7760 ± 0.8660	0 ± 0	0.0430 ± 0.0170
Cat ii	MI	Awake	2.8412 ± 1.2184	0.3056 ± 0.3844	0.0599 ± 0.0368
Cat iii	Parietal	Awake	3.1410 ± 0.8720	0.2010 ± 0.3680	0.0270 ± 0.0180
Cat iii	Parietal	SWS	4.2110 ± 0.7930	0.3290 ± 0.3620	0.0350 ± 0.0140
Cat iii	Parietal	REM 1	3.3240 ± 0.8150	0.2990 ± 0.2170	0.0290 ± 0.0110
Cat iii	Parietal	REM 2	3.4050 ± 0.8250	0.4250 ± 0.4470	0.0230 ± 0.0140
Human i	Temporal	Awake	3.5490 ± 0.8790	0.3870 ± 0.3650	0.0210 ± 0.0080
Human i	Temporal	SWS 1	3.6340 ± 0.6410	0.3790 ± 0.3150	0.0250 ± 0.0100
Human i	Temporal	SWS 2	3.2550 ± 0.5770	0.1710 ± 0.2670	0.0330 ± 0.0150
Human i	Temporal	REM 1	3.3740 ± 0.8560	0.0930 ± 0.1720	0.0300 ± 0.0090
Human i	Temporal	REM 2	3.6430 ± 0.5540	0.0960 ± 0.1950	0.0320 ± 0.0170
Human i	Temporal	Awake	3.9200 ± 0.7970	0.0080 ± 0.0230	0.0090 ± 0.0070
Human i	Temporal	SWS	3.8950 ± 0.7630	0.0070 ± 0.0140	0.0100 ± 0.0070

*Cross species summary of spike avalanche*.

**Table 2:** **Detailed awake spike avalanche**.

Loc	Bin size (ms)	CDF exponent	Pval	gof
MI	1	2.5	0	0.036
MI	3	5	0.008	0.020
MI	5	3.36	0	0.029
MI	7	3.63	0	0.039
MI	9	3.03	0	0.047
MI	11	3.83	0.327	0.034
MI	13	3.35	0	0.060
MI	15	2.83	0	0.089
PMd	1	4.1	0	0.006
PMd	3	2.81	0	0.021
PMd	5	5	0	0.018
PMd	7	4.85	0.061	0.017
PMd	9	4.03	0	0.022
PMd	11	4.21	0.018	0.024
PMd	13	4.25	0.216	0.019
PMd	15	4.08	0.61	0.017

*Monkey i detailed table*.

**Figure 3:**
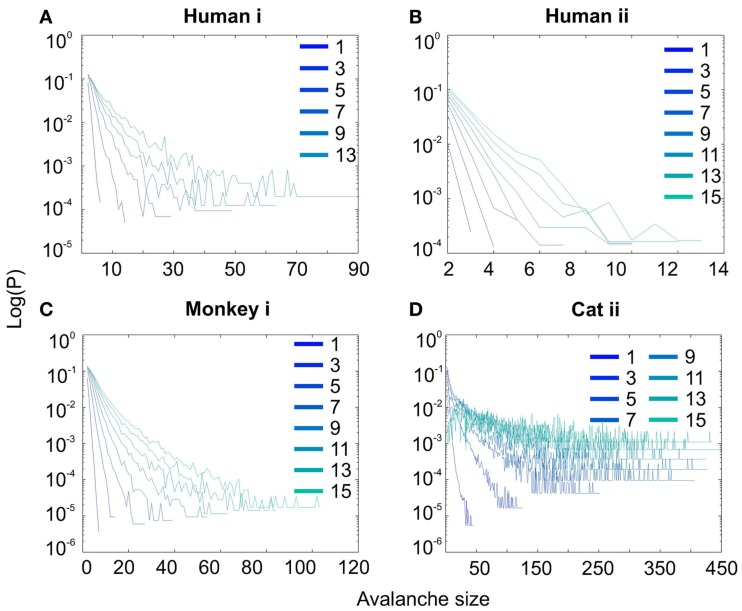
**Spike avalanche distributions in log-linear representation**. **(A–D)** Show results for different subjects. Different line colors refer to different bin sizes as shown in the legend. An exponential process has a linear trend in log-linear scale. Spike avalanches for all coarse graining levels, showed a linear trend. Please notice that bin sizes 11 and 15 are not shown because for the clarity in the line plot, but showed similar linear trend in this scale (not shown).

Interestingly, representing the size distributions in log-linear scale revealed a scaling very close to linear for all species (Figure 3), indicating that avalanches defined from spikes scale close to an exponential, as would be predicted by a Poisson type stochastic process. This conclusion was also reached previously by analyzing units and LFP recordings in cats (Bedard et al., 2006). Also, as can be seen in the inset of Figure 2A, the same analyses done on the awake recording from the parietal cortex (albeit spatially sampled at only 8 electrodes) shows similar scaling behavior.

In addition to wake resting recordings, we also considered recordings made while monkeys engaged in cognitive motor tasks. Similar to awake resting recordings in cat and man, the lower bound was variable between different binning sizes, thus excluding parts of the “invalid” initial avalanche sizes, which are usually used as evidence of existence of power-law (Beggs and Plenz, 2003; Petermann et al., 2009; Klaus et al., 2011). The inclusion of these initial parts may cause errors, and were removed here; however, their cumulative effects are still present in the tested regimen above *X_min_* of the analyzed “cumulative distribution function” (Goldstein et al., 2004; Newman, 2005; Bauke, 2007; Clauset et al., 2009). Above the lower bound value, all the CDF curves showed significant high exponent values. Interestingly, the MI (in both monkeys A and B) had similar mean to PMd (Table 1; Figures 2D–F), suggesting similar dynamics in the two areas.

#### Avalanche analysis during natural sleep

**Figure 4:**
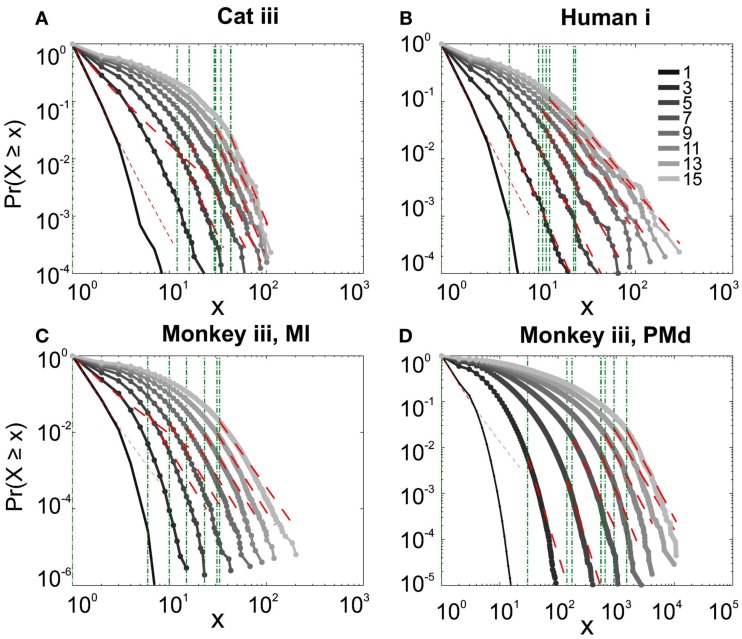
**Avalanche analysis of spiking activity during slow-wave sleep**. **(A)** Cat iii, **(B)** Human i, **(C)** monkey iii MI, and **(D)** monkey iii PMd. Different line colors refer to different bin sizes as shown in the legend. In parallel to awake dynamics (Figure 2), there is no sign of criticality, the curves follow different partial power-law with high exponents and variable lower bound values. The avalanche dynamics do not show a state-dependent trend. For the mean/STD exponent values, see Table 1.

**Figure 5:**
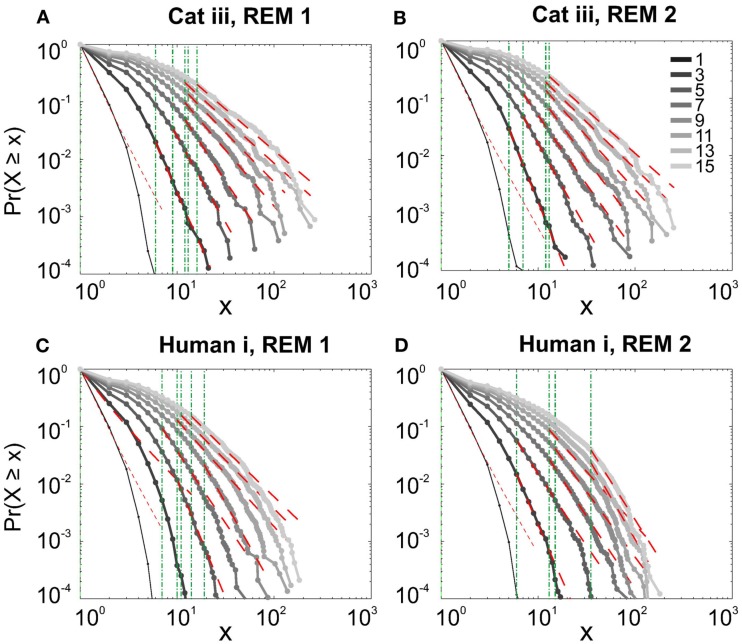
**Avalanche analysis of spiking activity during REM sleep**. **(A)** Cat iii REM episode 1, **(B)** cat iii REM episode 2, **(C)** human i REM episode 1, **(D)** human i REM episode 2. Different line colors refer to different bin sizes as shown in the legend. Similar to awake and SWS, the lack of criticality, variability through different coarse graining thresholds, and lower bounds is the universal finding. For the mean/STD exponent values, see Table 1.

It has been claimed that wakefulness may not be the best state to display SOC, and that avalanches may be more naturally related to brain states with oscillations, and slow-wave oscillations in particular (Hahn et al., 2011). In contrast to this, a previous study in cat found that like wakefulness, slow-wave sleep (SWS) did not display power-law scaling as defined from spike avalanches (Bedard et al., 2006), but this latter study suffered from a limited spatial sampling. To further investigate the issue, we have examined SWS and Rapid Eye Movement (REM) sleep periods with more dense sampling of spike activity. Figures 4 and 5, show the analyses for cat, human i and ii as well as monkey iii (MI and PMd) for SWS and REM periods. In none of these cases we, see clear sign of power-law scaling. In all cases (except human ii), the variability of lower bound between different bin sizes is robust. All the curves represent “partiality of power-law” with high exponent values. During SWS, cat, human subjects, and monkey iii (MI and PMd) all manifested either lack of significant power-law scaling, or had such higher exponent values that makes it highly unlikely for power-law to be the generating process of spike dynamics (Table 1). Similarly, in REM periods, there was no evidence for power-law scaling in human i’s first and second REM episodes. Together, with Cat REMs’ high exponents values, power-law scaling appears to be an unlikely candidate to describe the statistics of neural firing (Table 1). Taken together, these various tests all based on proper statistical inferences, show that spike avalanches do not follow power-law scaling, for any brain state or sampling density.

Detailed numerical values for spike avalanche CDF exponents and their goodness of fit are provided in Tables 1 and 2.

### Avalanche dynamics from local field potentials

Next, we investigated the occurrence of avalanche type of dynamics from the local field potentials, which were simultaneously recorded with unit activity, in all datasets.

#### Relation between LFP peaks and spiking activity

**Figure 6:**
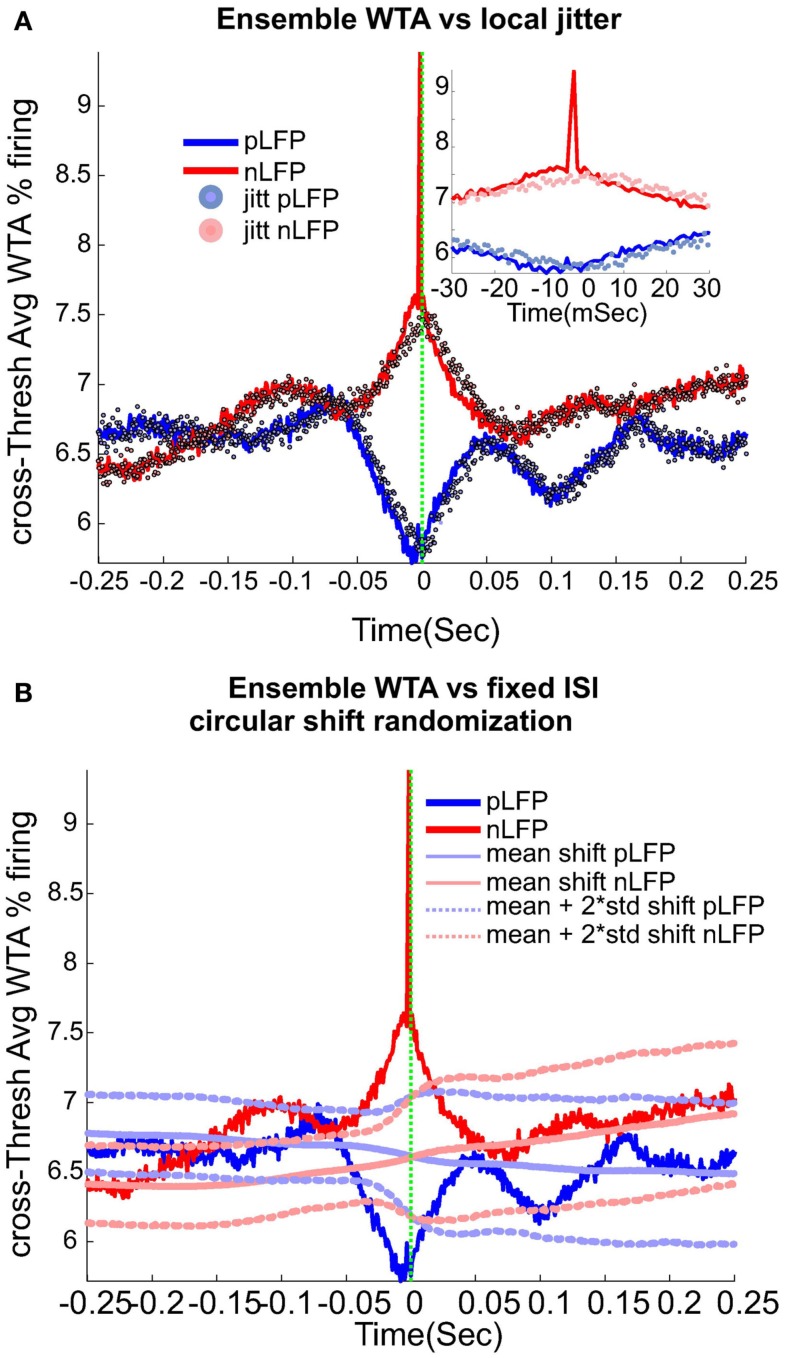
**Relation between unit firing and LFP peaks in wakefulness**. nLFP (red) and pLFP(blue)-based wave-triggered-average (WTA) of percentage unit activity, showing that the negative peaks have closer association with an increase of neuronal firing. **(A)** Tightly regulated local jitter of nLFP peaks destroys the large nLFP peak. Inset shows the zoom around 0. **(B)** Preserving the internal structure of aggregate spike train and ensemble LFP peaks, but destroying the relation between the two leads to the disappearance of the nLFP peak. See text for details of randomization and controls. The WTA traces in this figure are from Human i, (based on 183127, 98520, and 47451 nLFP and 158737, 79225, and 36020 pLFP peaks for low, medium, and high threshold respectively.)

Calculation of neuronal avalanches from LFP data is based on the assumption that statistically speaking, in comparison with the positive LFPs (pLFP), the negative LFP (nLFP) peaks are more strongly related to neuronal activity (e.g., see Destexhe et al., 1999 and references therein). Indeed, the 8-electrode cat LFP data analyzed here show such a relation (Destexhe et al., 1999; Touboul and Destexhe, 2010). To further test this relation, we also examined the simultaneous LFP and unit recordings in the ensemble recordings in cat, man, and monkey. We used a wave-triggered-average (WTA) procedure, where the ensemble of nLFPs were used to epoch the ensemble spike activity. Averaging across these WTAs across different thresholds, show that there is indeed a weak relationship between nLFP and spiking (Figure 6A). However, repeating the same procedure for positive LFP (LFP) peaks, did not display any relation (Figure 6B), in agreement with the same analysis in cats (Touboul and Destexhe, 2010). Through four different types of control and randomization, we show that the relation between nLFP and spike is robust and is not attributable to randomness of the spiking events or spurious fluctuations in the LFPs. For details of these control/randomization, see methods and Figure 6. This fundamental difference between nLFP and pLFP peaks provides a very important test to infer if a given power-law observation from LFPs is related to the underlying neuronal activity, as we will, see below.

#### nLFP avalanches

**Figure 7:**
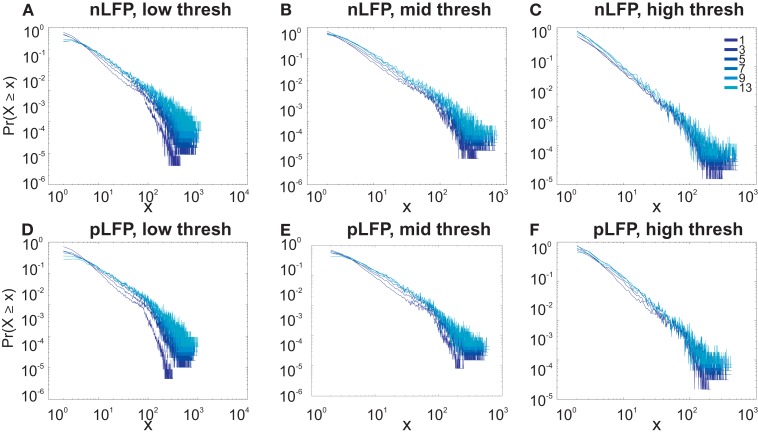
**Avalanche analysis in awake monkey LFPs in logarithmic representation**. A power-law process has a linear trend in log-log scale. LFP (negative or positive) maxima avalanches for all coarse graining levels, as well as all thresholds, showed a linear trend. Upper row **(A–C)**, shows the nLFP for low, mid, and high thresholds respectively. Lower row **(D–F)**, shows the same for pLFP. Please notice that bin sizes 11 and 15 are not shown because for the clarity in the line plot; however, they too, also showed a very clear linear trend in this scale. Such trend is necessary but not sufficient for a process to be power-law. See text and Figure 9.

**Figure 8:**
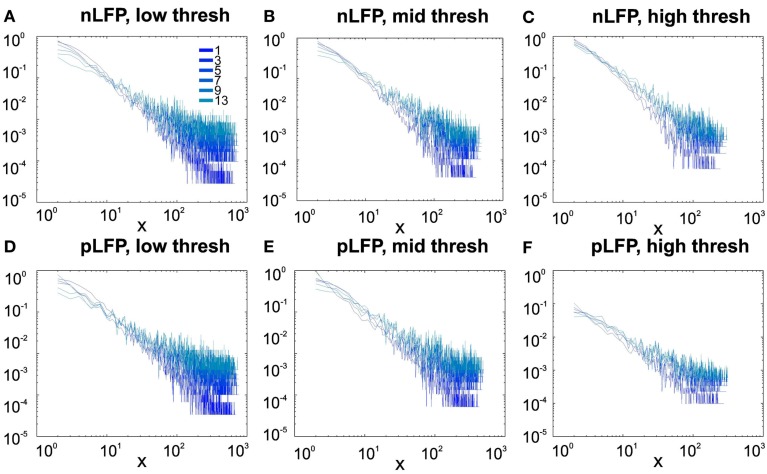
**Avalanche analysis in awake human LFP in logarithmic representation**. A power-law process has a linear trend in log-log scale. LFP (negative or positive) maxima avalanches for all coarse graining levels, as well as all thresholds, showed a linear trend. Upper row **(A–C)**, shows the nLFP for low, mid, and high thresholds respectively. Lower row **(D–F)**, shows the same for pLFP. Please notice that bin sizes 11 and 15 are not shown because for the clarity in the line plot; however, they too, also showed a very clear linear trend in this scale. Such trend is necessary but not sufficient for a process to be power-law. See text and Figure 9.

**Figure 9:**
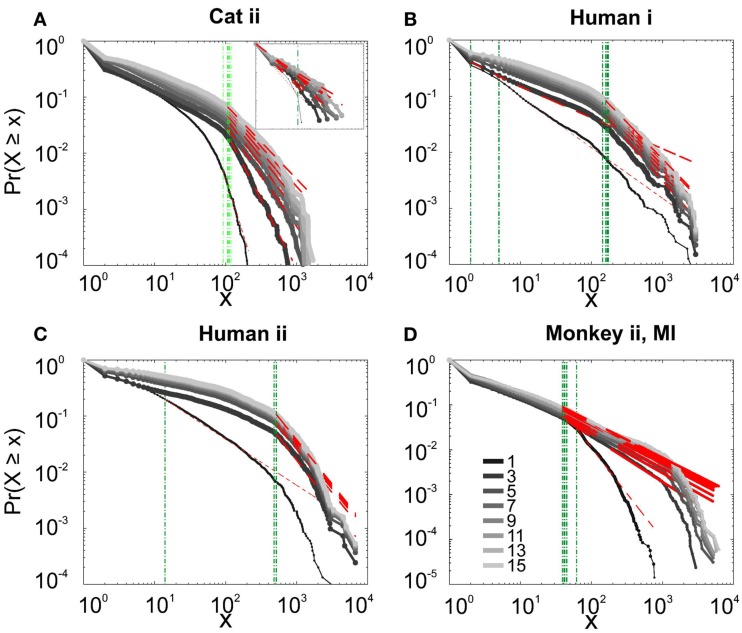
**Avalanche analysis based on LFP negative peaks in wakefulness**. **(A)** Cat ii (96 electrode array) and Cat iii (inset, 8 electrode array), **(B)** Human i, **(C)** Human ii, **(D)** Monkey ii MI. In all cases, different binnings lead to variable lower bound and scaling exponents. Lack of linear trend in CDF shows that the observed linear trend in log-log scale, as shown in Figures 7 and 8, are not sufficient for showing that avalanche dynamics are power-law processes. For the mean/STD exponent values, see Table 3.

Similar to previous studies, we investigated the avalanche dynamics from nLFPs. The nLFPs were detected using a fixed threshold, defined as a multiple of the standard deviation (STD) of the LFP signal (see Methods), and several thresholds were tested. In the following, we use “high,” “medium” and “low” thresholds, which correspond to 2.25, 1.75, and 1.25 multiples of the standard deviation, respectively. As shown in Figures 7 and 8, the distributions defined for avalanches at different bin sizes and thresholds seem to display power-law scaling, both for human and monkey. This result seems to be in agreement with similar analyses done on awake monkey (Petermann et al., 2009). However, plotting the same data as CDF revealed that the scaling as power-law was very narrow (Figure 9). While Monkey ii displayed apparent power-law over more than one decade, the other cases from cats and humans, did not display any convincing power-law scaling. For details of nLFP avalanches for an example subject, and its comparison with pLFP avalanches, see Table 3. One can also note that in some of the CDFs (and their counterpart PDF), there is a possibility that the distribution can be segmented into two regions each covering certain decades of avalanche size. In such cases, relying on a single scaling exponent to describe the totality of the functional dynamics of the network does not seem adequate. This could be an indication that the space of the distributions is not uniform and the underlying mechanisms could be of metastability nature (Mastromatteo and Marsili, 2011). In such scenario, interaction with the external world could push the system from the “currently most stable state” to a new “most stable state.” Such constant changes may lead to the formation of non-uniform distribution of the neural events at different temporal scales. Therefore it is essential to emphasize that, in some cases, one scaling exponent may not be sufficient to describe the complexity of the spiking or oscillations.

**Table 3:** **Detailed awake LFP avalanche**.

Bin size (ms)	Polarity	Threshold	CDF exponent	Pval	gof
1	Neg	Low	1.71	0	0.019
3	Neg	Low	2.99	0.056	0.051
5	Neg	Low	2.55	0	0.052
7	Neg	Low	2.84	0.074	0.052
9	Neg	Low	2.42	0	0.053
11	Neg	Low	2.37	0	0.059
13	Neg	Low	2.43	0	0.054
15	Neg	Low	2.36	0	0.052
1	Neg	Mid	1.83	0.002	0.015
3	Neg	Mid	2.79	0.425	0.040
5	Neg	Mid	2.84	0.55	0.042
7	Neg	Mid	2.81	0.376	0.048
9	Neg	Mid	2.84	0.345	0.050
11	Neg	Mid	2.84	0.435	0.048
13	Neg	Mid	2.71	0.098	0.058
15	Neg	Mid	2.74	0.204	0.056
1	Neg	High	1.9	0	0.018
3	Neg	High	1.55	0	0.029
5	Neg	High	2.44	0.645	0.036
7	Neg	High	2.43	0.201	0.046
9	Neg	High	2.41	0.672	0.036
11	Neg	High	2.39	0.67	0.035
13	Neg	High	2.3	0.496	0.036
15	Neg	High	2.3	0.36	0.040
1	Pos	Low	1.68	0	0.020
3	Pos	Low	1.37	0	0.073
5	Pos	Low	3.03	0	0.066
7	Pos	Low	4.21	0.762	0.051
9	Pos	Low	3.59	0.585	0.048
11	Pos	Low	3.39	0.43	0.047
13	Pos	Low	2.98	0.079	0.046
15	Pos	Low	2.9	0.032	0.052
1	Pos	Mid	1.74	0	0.018
3	Pos	Mid	3.67	0.128	0.062
5	Pos	Mid	3.79	0.047	0.069
7	Pos	Mid	5	0.827	0.061
9	Pos	Mid	3.78	0.797	0.041
11	Pos	Mid	3.68	0.926	0.036
13	Pos	Mid	3.87	0.797	0.049
15	Pos	Mid	3.51	0.553	0.046
1	Pos	High	1.76	0.009	0.020
3	Pos	High	1.47	0	0.061
5	Pos	High	3.19	0.169	0.067
7	Pos	High	3.17	0.063	0.066
9	Pos	High	3.07	0.251	0.061
11	Pos	High	3.09	0.325	0.059
13	Pos	High	3.18	0.286	0.062
15	Pos	High	2.74	0.033	0.061

*Human i detailed Table*.

#### pLFP avalanches

**Figure 10:**
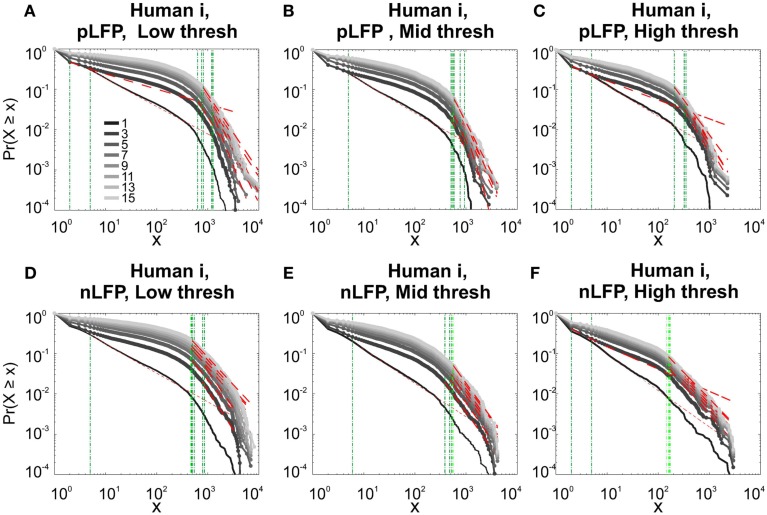
**Comparison of Avalanche analysis based on negative and positive peaks**. **(A–C)** Show the CDF for different thresholds of pLFP and **(D–F)** are related to nLFP. LFP (negative or positive) maxima avalanches for all coarse graining levels, as well as all thresholds did not show linear trend in CDF, therefore negate power-law as the generating process. These curves show while nLFP has a closer relation with spiking, the avalanche dynamics of nLFP and pLFP are strikingly similar in their lack of robust criticality when tested with rigorous statistical tests.

Next, we investigated the avalanche dynamics of positive LFP peaks, which, as we have seen above, is not statistically related to firing activity (Figure 6). Similar to nLFP peaks, the pLFP avalanches defined for human wakefulness did not display power-law scaling (Figure 10). Both nLFP and pLFP had similar CDF of avalanche size across different species and cortices. The example shown in Figure 10 (awake human) shows that across different thresholds, both nLFP and pLFP had variable lower bounds and high scaling exponents for the region of the data that could statistically be considered for power-law properties. Moreover, the absence of any region with clear linear scaling in the logarithmic coordinates further confirms that there is no power-law scaling in this case. For details, see Table 3.

#### Avalanches in different cortical regions

**Figure 11:**
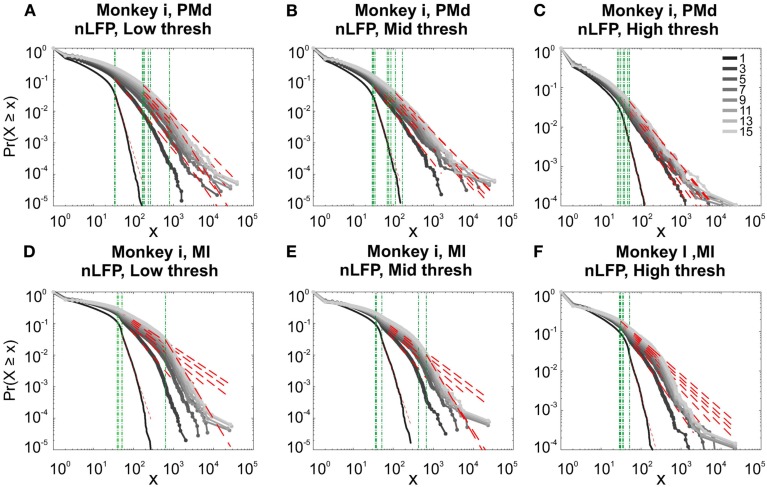
**Avalanche analysis in different cortical areas recorded simultaneously**. Avalanche dynamics in nLFP shows that the CDF of the input and output units of two interacting cortices have slightly different characteristics but neither follow criticality regime. **(A)** Monkey i, MI, low threshold **(B)** Monkey i, MI, medium threshold, **(C)** Monkey i, MI, high threshold, **(D)** Monkey i, PMd, low threshold **(E)** Monkey i, PMd, medium threshold, **(F)** Monkey i, PMd, high threshold.

In the cases that we had simultaneous, dual array multielectrode recordings from PMd and MI, the analyses showed that these two cortical areas do not show signs of criticality but have slight differences in their exponent values for MI and for PMd (Tables 1 and 2; Figure 11). Such findings show that the fact that these two cortices directly interact with each other, and one acts as input and one as the output of motor processing unit, is reflected in their slightly different CDF features. Thus, two different cortical areas seem to display similar features, although no sign of power-law scaling.

### Statistical analysis of the avalanche distributions

#### Goodness of fit

Given data *x* and given lower cutoff for the power-law behavior *X_min_*, we computed the corresponding *p*-value for the Kolmogorov-Smirnov test, according to the method described in Clauset et al. (2009). See methods for details. The results are given in Tables 1, 2, and 3 (“gof” columns).

#### Avalanche size boundaries

Imposing lower or upper bounds when fitting avalanche distributions can greatly affect the outcome of the fit (Clauset et al., 2009). In many cases, the analyses have been limited to the lower boundary of avalanche size = 1 and *X_max_* of N, where N is the number of channels. Using such bounds improves the fitting of the data by power-law compared to other distributions, as confirmed by KS-statistics (Klaus et al., 2011). The pitfalls of such an approach are two-fold: (a) the lower boundary is set to 1, therefore the avalanches that are below the acceptable lower bound of *X_min_* are erroneously fitted with the power-law, thus reducing the reliability of the fit while producing mis-estimated scaling exponents (see Clauset et al., 2009 for details of lower bound selection). (b) *X_max_* is set to the maximum active channels, and any return to a given channel is counted in the avalanche, but the maximum allowed avalanche size is limited to N, based on the argument that the large avalanches are infrequent and their inclusion implies misfit. This type of approach, limits the number of avalanches to an extreme degree and introduces a bias. Below we investigate this bias.

#### Avalanche size distribution and upper boundary limits

**Figure 12:**
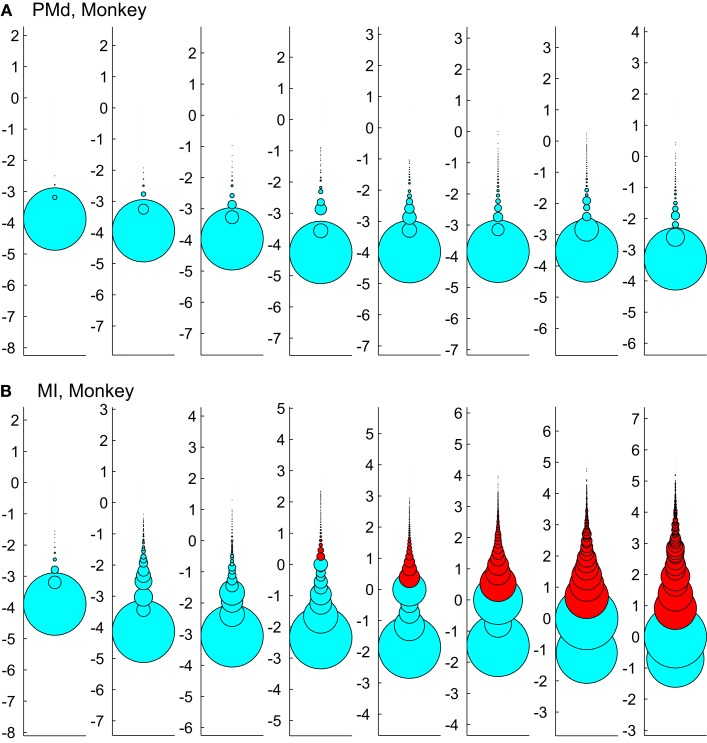
**Effects of setting upper boundaries on avalanche size distribution**. Each column shows avalanches of a different bin size (increasing from left to right). **(A,B)** Show the results of spike avalanche size distribution of the PMd and MI (respectively). For each bin size, the distributions of different avalanche sizes are shown in circles; the avalanche size increases from the bottom to the top, while the size of each circle represents the ratio to the overall number of avalanches. Red color shows the excluded (saturated) avalanches enforced by limiting the *X_max_* to N (number of independent measures; i.e., units in the case of spike avalanches and electrodes in the case of LFP avalanches). Cyan color shows the included avalanches. Y axis is in logarithmic scale for better visualization and the values of Y represent the orders of magnitude of N for proper comparison between different bin sizes (i.e., a given circle at y = 2, represents the avalanches that their size = 2 log(N), its diameter shows the number of avalanches that had that size and its color shows whether it is included or excluded according to the *X_max_* = N rule).

Figure 12 tests the effect of enforcing an upper boundary to the avalanche analysis. The red color shows the excluded (saturated) avalanches enforced by limiting the *X_max_* to N (number of independent measures), while cyan represents the acceptable avalanches below this upper threshold. This figure shows that setting the *X_max_* to a cutoff value of N, produces variable biases based on the bin size. Importantly, in simultaneously recorded regions, the majority of avalanches will be included in one case (like in PMd as shown in Figure 12A) but not in the other (like MI, as depicted in Figure 12B). Such discrepancy emphasizes that setting a cutoff will necessarily introduce a bias and causes variable results from region to region and from bin size to bin size.

#### Comparison of exponential and power-law fit: model mis-specification and lower boundary problem

**Figure 13:**
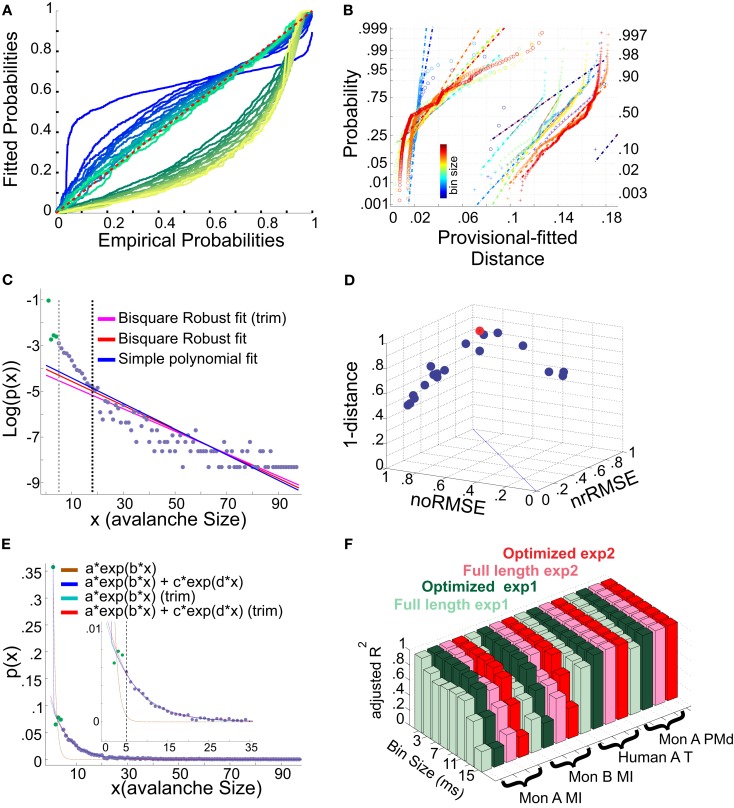
**(A,B)** Fits comparison and lower boundary. **(C–F)** Alternative fits for avalanche size distributions. **(A)** Probability-Probability plot (ECDF vs. provisional CDF) for a sample bin size (cat i spike avalanche). Green colors are p-p plot for ECDF vs. exponential, and blue colors are for p-p plot for ECDF vs. power-law. In each color family, as the lower boundary is increased (from 1 to *X_min_*), the color saturation fades; i.e., darkest color shows lower boundary of 1 and the lightest shows lower boundary of *X_min_* (where *X_min_* is based on the Clauset method for fitting power-law to empirical data). **(B)** Integral of p-p distance to the 1:1 diagonal (perfect match of the parametric CDF to ECDF). The colors (blue to red) are related to bin sizes (from smallest to biggest). Cross signs represents exponential distance and circles represents power-law distance to the ECDF. **(C)** Simple exponential fitting of spike avalanche data. The data points (purple and green) are plotted in a log-linear representation, together with a simple polynomial fit (blue), a robust fit calculated on the full length data (red) and a robust fit on the reduced data (magenta). The two vertical lines indicate the lower bound of the region of linearity, i.e., “*X_min_*,” calculated based on the simple polynomial fit (black) and the bi-square method (gray). **(D)** Comparison of the goodness of fit of different exponential fits to different reductions of the same dataset. The 3 coordinates are “normalized overall improvement of RMSE” (noRMSE), “normalized relative improvement of RMSE” (nrRMSE) and distance of a point from the diagonal in (noRMSE,nrRMSE) plane. Each point in this 3D space, is the result of a bi-square robust fit after elimination of the first *i* elements of the data (best fit in red). **(E)** Bi-exponential fitting of the same data. The “sum of exponential” model (exp2) gave a very good performance in both full length (dark blue) and reduced above “*X_min_*” (red). The “simple exponential” model (exp1) reaches a very good fit only for the reduced set (cyan) but not for the full length of the avalanches (light brown). **(F)** Effects of linearity improvement on exponential fits. Each set of four colors refer to the spike avalanche of Monkey i (MI), Monkey ii (MI), Human A(Temporal), and Monkey i (PMd). In each set, green colors refer to the simple exponential family (exp1) and the red colors depict the sum of exponentials (exp2). Light green and light red, refer to the calculated ${\bar{R}}^{2}$ on full length avalanche sizes, while dark green and red show the average ${\bar{R}}^{2}$ for the dataset ranging from *N* − 1 to *N* − *X_min_* where the optimized length *X_min_* was 5 [see **(C,D)**]. **(C–E)** Were obtained from 15 ms bin avalanches from human i awake spikes.

It has been argued whether neuronal avalanches are better fitted by an exponential or power-law distribution. Here we tested two aspects, exponential vs. power-law comparison, as well as the effect of setting a lower boundary to the fit. It has been shown that defining a proper lower boundary improves the maximum likelihood that the distribution could be fit by a power-law (Clauset et al., 2009). In agreement with this, Klaus et al. (2011) used a lower boundary of 1 and showed that using KS-statistics, the power-law indeed provides a better fit to the data in comparison to exponential distribution. Here, we systematically tested whether such practice would return erroneous results in avalanche analysis. The results shown in Figures 13A,B, are from cat spikes data. For each bin size, we first defined the optimal lower boundary after Clauset et al., 2009; see Methods), called *X_min_*. We started with a lower boundary set to 1, and reduced the distribution of avalanche data gradually up to *X_min_*. For each newly produced set, we calculated the empirical CDF (ECDF) as well as the provisional fitted probability’s CDF (based on direct maximum likelihood) for both exponential as well as power-law. The results for a sample bin size are shown in Figure 13A. Power-law at the lower boundary of 1 provides a bad fit. However, overall, power-law outperforms the exponential fit, specially after limiting the range of the data by increasing the lower boundary. The best power-law fit is obtained when the lower boundary approaches *X_min_*.

This finding matches the results of the KS test (based on Clauset et al., 2009) as we report in this manuscript. However, from our analyses, we know that when we reach the best power-law fit, the estimated scaling exponents are too high for any known natural system to follow a self-organized criticality regime. Therefore, we have a situation where either one gets unreliable but desired scaling exponent by setting the lower boundary to 1, or one obtains reliable but undesired scaling exponent by setting the lower boundary to *X_min_* > 1.

Next, we quantified the goodness of fit with a more rigorous approach than the simple KS test. If the parametric CDF is close to the probabilities from the ECDF, then the depicted line should approach the diagonal (1:1) line with minimal drift from it. For quantifying this, we measured the integral of the distance of each point on the p-p curves from the 1:1 diagonal line. This value should be zero in a perfect fit; its non-zero value shows departure from a perfect fit. Figure 13B shows the results for all bin sizes. Similar to Klaus et al. (2011), the power-law provides a better fit in comparison to exponential. However, there are two aspects that can not be ignored for this condition to be true: (a) the distance improves only as we tighten the lower bound criteria to be close to *X_min_*, but it does not mean that this is a proper fit; (b) there is no rule of thumb for such an improvement; in almost all of the cases, a linear relationship in the normal probability plot distribution of the distance was not found. This shows that power-law provides a better fit than the exponential distribution, but that both fits are not satisfactory. We consider alternative distributions below.

#### Alternative distributions for avalanche dynamics

Although previously, at the microcircuit scale, some studies have asserted the existence of criticality as a universal characteristic of neural dynamics in both spike and LFP avalanches (Beggs and Plenz, 2003; Ribeiro et al., 2010), other evidence suggest that same behavior can also be observed through stochastic processes (Bedard et al., 2006; Touboul and Destexhe, 2010). In this study, after rigorous testing, we showed that the avalanches do not follow power-law as a universal feature. Thus we also tested whether an alternative probability distribution could provide a better estimate for the experimental observations.

We first tested a simple exponential fitting of the spike avalanches, by fitting straight lines in a log-linear plot. As seen from Figure 13C, a linear fit (“exp1”) can only fit part of the data, as the initial points (for small size) do not scale linearly. In detection of the lower bound of linearity, i.e. (*X_min_*), the robust bi-square method is more stringent than simple least square fits and leaves behind more data points for exponential fitting (see different lines in Figure 13C; errors based on bi-square are plotted in Figure 13D; see Methods for details on linearity optimization).

Next, we tested a multiple exponential fitting of the data. The rationale is that two exponential processes may represent differences in two populations of cells, for example excitatory and inhibitory cells. The fit resulting from a “sum of exponential processes” was extremely good in minimum residual and reliable prediction bounds for the data (Figure 13E). This “sum of exponential” model (“exp2”) gave a very good performance in both full length (dark blue) and reduced above “*X_min_*” (red). The “simple exponential” model (exp1) reaches a very good fit only for the reduced set (cyan) but not for the full length of the avalanches (light brown). For comparison of “exp1” and “exp2” on different spike avalanches, with and without “linearity improvement,” see Figure 13F. Overall, it seems that both exp1 and exp2 exhibit comparably high values of goodness of fit for the reduced sets. However, only the double exponential fit was able to fit the entire dataset.

## Discussion

In the present paper, we have analyzed and compared the avalanche dynamics obtained from multielectrode recordings of spikes and LFPs, for three species, cat, monkey, and human. In each case, we used recordings exclusively made in non-anesthetized brain states, including quiet and active wakefulness, SWS (slow-wave sleep), and REM (Rapid eye movement). The primary result of our analysis is that there is no power-law scaling of neuronal firing, in any of the examined recordings, including “desynchronized” EEG states (wakefulness), SWS, and REM sleep. All species consistently showed distributions which approached exponential distributions. This confirms previous findings of the absence of power-law distributions from spikes in cats (Bedard et al., 2006), and extends these findings to monkeys and humans. An obvious criticism to that prior study is that a set of 8 electrodes is too low to properly cover the system, and the absence of power-law may be due to this subsampling. We show here that the same results are obtained when a significantly higher density of recording is used, confirming the absence of power-law.

In contrast, avalanche dynamics built from nLFPs displayed more nuanced results. In some cases, the avalanche size distributions appear to draw a straight line in log-log representations, but the more reliable CDF based tests did not show clear evidence for power-law scaling. Indeed, statistical tests such as the KS test did not give convincing evidence that these data are universally distributed according to a power-law. More importantly, while nLFP are related to firing activity, we showed that a similar behavior was also observed for pLFP peaks. The avalanche analysis from positive peaks displayed similar results as for negative peaks, although positive peaks displayed a weaker statistical relation to firing activity. Using 4 types of control/randomization we provide very robust evidence that the fundamental differences between nLFP and pLFP are not attributable to random behavior of spikes or LFP peaks. Yet still, the discretized thresholded LFPs, show strikingly similar behavior in their avalanche statistics. These findings render any conclusions about self-organized criticality based on simple power-laws of PDFs as phenomenological.

Together, these results suggest that the power-law behavior observed previously in awake monkey (Petermann et al., 2009; Ribeiro et al., 2010) cannot be reproduced in awake humans’ temporal cortex or cat and monkey motor cortex. This conclusion also extends to slow-wave sleep and REM sleep, which we found did not display power-law distributed avalanches, as defined from either spikes or LFPs. In searching for the linear domains in CDF based on the KS test, one can force the scaling exponent to fall within the range of the plausible values (comparable to those observed in known physical phenomena). Doing so, of course, yields more conservative values of scaling, but means that such scaling would be applicable to only a limited range of data. In fact, unless the system has universal scaling, there is always a tradeoff between the range to which a scaling exponent can be extended (i.e., the linear regime in the data) and the proximity of the scaling exponent value to those of a narrow range (in this case, values of the SOC systems are of interest). Our tests, did not force the scaling exponent to be limited to values between 1 and 2, therefore it had a more stringent emphasis on the linearity of more decades of the avalanche sizes. In some cases where the data showed statistically significant linearity, the obtained scaling exponents were an order of magnitude higher than what falls in the range of the critical regime of known physical phenomena. Conversely, these observations imply that, a single scaling exponent is not sufficient to explain the complex dynamics of ensemble activity.

A possibility worth exploring is that some form of power-law in LFPs is the result of volume conduction associated with LFPs recorded in high density arrays. When a peak is detected, it is often also present in many different channels. A possibility worth to explore is whether the same event could be volume-conducted across many channels in the array, which may lead to an artificial increase the large-size avalanches. This possibility should be examined by mathematical models of the volume conduction effect.

It must be noted that the evidence for self-organized criticality in neuronal cultures or in slices (Beggs and Plenz, 2003), as well as in anesthetized states (Hahn et al., 2010) is not contradictory with the present findings. The wiring of *in vitro* preparations, as well as the network dynamics in anesthesia, are evidently different than in the intact brain (Steriade, 2001). We find here that there is no evidence for SOC in wakefulness and natural sleep states, and for 3 different species. On the other hand, the report of power-law scaling of nLFPs avalanches in awake monkey (Petermann et al., 2009) seems in contradiction with the present findings. Many possibilities exist to reconcile these observations, such as differences between brain region, recording method, cortical layer, or volume conduction effects. These possibilities should be investigated in future studies. Moreover, in a recent report (Friedman et al., 2012), it has been shown that data from high density recordings (up to 512 electrodes) from from neural culture show elements of universality and that avalanches can be collapsed into a universal scaling function (Papanikolaou et al., 2011). Such findings confirm that brain circuits *in vitro* operate near criticality. Further studies should examine how to reconcile such evidence with the present *in vivo* findings.

Due to the high dimensionality of neural data, it is crucial to separate the features of the inferred models that are induced solely by the inference scheme from those that reflect natural tendencies of the studied system (Mastromatteo and Marsili, 2011). In some cases, one could fit the data with different lines by limiting the range of the decades within which a fit is analyzed. While it is indeed possible, and highly likely, that neural data at this level follow a multi-scale regime, albeit such a property would push the system away from cohesively operating at self-organized criticality because the relation between microscopic interaction of the (neural) elements and collective behavior (of the cortical network) no longer manifests in single valued features, like a single scaling exponent.

Finally, it is important to emphasize that the present results were obtained using statistical tests similar to previous statistical analyses (Newman, 2005; Clauset et al., 2009). In particular, the use of the CDF distribution rather than simple log-log representations of the size distribution is a particularly severe test to identify if a system scales as a power-law. The use of statistical measures such as the Kolmogorov-Smirnov test (Tables 1, 2, and 3) also constitutes a good quantification of which distribution fits the data, and is largely superior to the least square fit in double logarithmic scale (Clauset et al., 2009). The uncertainty and goodness of fit were estimated by 1000 repetitions of each fitted distribution. We also showed that setting bounds to the fit can introduce biases in favor of power-law fits, as analyzed previously (Clauset et al., 2009). In agreement with this, it was found with bounded fits that power-law provides a better match to data compared to exponential distributions (Klaus et al., 2011). Our analysis shows that neither power-law nor exponential distributions provide acceptable fits to the datasets analyzed here. Multi-exponential fits suggest that bi-exponential processes provide a particularly good fit to the distributions, which suggests that the underlying neuronal dynamics is most compatible with two exponential processes, which could be for example excitation and inhibition, both scaling as exponential distributions. Such a possibility should be tested by further studies, and seem in agreement with the complementary excitatory and inhibitory dynamics found in the awake and sleeping brain (Peyrache et al., 2012).

## Conflict of Interest Statement

The authors declare that the research was conducted in the absence of any commercial or financial relationships that could be construed as a potential conflict of interest.
